# A transgenic rotifer-based RNA interference approach for antiviral protection against *Covert mortality nodavirus* in shrimp aquaculture

**DOI:** 10.3389/fmicb.2026.1860691

**Published:** 2026-06-10

**Authors:** Ming Ge, Fang Xu, Jinrui Tan, Siyu Huang, Songbei Ying, Yating Wang, Yumin Lv, Xinyao Yuan, Jiahan Zhang, Mingxu Li, Yijing Zhang, Hua Xu

**Affiliations:** 1 State Key Laboratory of Genetic Regulation of Complex Traits, School of Life Sciences, Fudan University, Shanghai, China; 2 Yellow Sea Fisheries Research Institute, Chinese Academy of Fishery Sciences, Qingdao, China

**Keywords:** *Brachionus plicatilis*, *Covert mortality nodavirus* (CMNV), *Penaeus vannamei*, piggyBac, RNAi

## Abstract

Viral diseases inflict severe economic losses on global aquaculture, driving the urgent need for sustainable and effective disease management strategies. In this study, we developed a feeding-based RNA interference (RNAi) strategy, utilizing the rotifer *Brachionus plicatilis* as a live delivery vehicle for short hairpin RNA (shRNA) against *Covert mortality nodavirus* (CMNV) to control covert mortality disease (VCMD) in *Penaeus vannamei*. A piggyBac-mediated transgenic platform was established in *B. plicatilis* based on genomic and transcriptomic resource development. Stable and heritable expression of a reporter gene was first demonstrated, confirming the feasibility of genetic manipulation in this non-model organism. CMNV was subsequently detected in farmed *P. vannamei*, and *CMNV-specific shRNA* expression cassettes were introduced into rotifers using endogenous promoter elements. Feeding experiments revealed that shrimp fed *CMNV-shRNA*–expressing rotifers exhibited a significant reduction in viral load, as determined by quantitative PCR. Under artificial CMNV challenge conditions, shrimp receiving transgenic rotifers showed reduced cumulative mortality compared with control groups. Importantly, no adverse effects on shrimp growth performance were observed. In summary, transgenic rotifers can serve as an effective and scalable live-feed platform for antiviral RNA delivery. This study provides a new concept for integrating RNAi-based disease control strategies into shrimp hatchery systems and offers a promising approach for sustainable management of viral diseases in aquaculture.

## 1 Introduction

Viral disease continues to be a major problem in shrimp aquaculture, and among the pathogens involved, *Covert mortality nodavirus* (CMNV) has attracted increasing attention. The virus was first linked to covert mortality disease in farmed shrimp back in 2014 (Zhang et al., 2014), and the condition was later given the name viral covert mortality disease (VCMD) to make its viral etiology explicit (Zhang et al., 2017). What makes CMNV particularly troublesome is how many hosts it can infect. Within crustaceans alone, the virus turns up in commercially important species, including *Penaeus vannamei*, *Penaeus monodon*, *Penaeus chinensis*, and *Penaeus japonicus* (Thitamadee et al., 2016), as well as in wild crabs (Zhang et al., 2017; Liu et al., 2018). Perhaps more alarming, several teleost fish species have also tested positive (Wang et al., 2019, 2021a; Xu et al., 2020), which points to a genuine capacity for jumping between taxonomic groups in shared aquatic environments. Most alarmingly, a recent landmark study has identified CMNV as a potential causative agent of an emerging human eye disease, persistent ocular hypertensive viral anterior uveitis (POH-VAU), marking the first evidence of an aquatic virus jumping to humans and causing direct pathology. This zoonotic risk, linked to the handling and consumption of infected aquatic products, elevates CMNV from a purely economic concern in aquaculture to a significant public biosecurity threat (Liu et al., 2026). Unlike acute shrimp viruses that usually cause rapid and obvious outbreaks, CMNV often persists as a chronic or subclinical infection (Zhang et al., 2017, 2018). This infection pattern complicates early diagnosis and limits the effectiveness of conventional disease management measures. The situation is compounded by the fact that no targeted antivirals or vaccines exist for shrimp at this stage (Gholamhosseini et al., 2020), and disease control strategies mainly rely on biosecurity management and pathogen screening, which are often insufficient under intensive farming conditions. Consequently, there is an urgent need to develop practical, sustainable, and aquaculture-compatible antiviral strategies for CMNV control.

Chemotherapeutic agents do offer a degree of infection control on shrimp farms, but the downstream consequences—particularly drug residues that may end up in consumer products—raise real concerns (Tomazelli et al., 2018). How effective antiviral chemotherapy actually is in aquaculture settings has itself been questioned (Boyd et al., 2007). Turning to vaccination is not a straightforward option either, given that shrimp, like other invertebrates, simply do not possess immunoglobulins or any comparable adaptive immune architecture (Satoh et al., 2008). This biological reality rules out the conventional vaccine strategies that work so well in finfish. One mechanism that does hold genuine promise is RNA interference (RNAi). RNAi sits at the heart of innate antiviral defense in invertebrates (Robalino et al., 2005) and works by degrading viral RNA in a sequence-specific manner (Fire, 2007). Multiple studies have now shown it can protect aquatic animals from viral challenge (Deng et al., 2014). The catch, though, is that laboratory success has not easily translated to the farm. Free RNA molecules break down quickly once exposed to pond water, getting them into the right cells and tissues is still an unsolved problem, and nobody has yet found a reliable way to make the antiviral effect last long enough to matter across a full grow-out cycle. Until these practical obstacles are addressed, RNAi will remain a tool of the laboratory rather than the hatchery.

Live feed organisms provide a unique opportunity to overcome these limitations by acting as biological carriers for RNAi delivery (Charoonnart et al., 2023). Marine rotifers of the genus *Brachionus* are indispensable live feed during the early developmental stages of shrimp and are routinely used in shrimp hatcheries worldwide. Specifically, *Brachionus plicatilis* is mass-cultured globally due to its rapid population doubling times, euryhaline adaptability, and low production costs (Lawrence et al., 2012; Cahu and Infante, 2001). Because postlarval and juvenile shrimp graze continuously on these rotifers, bioencapsulating antiviral effectors within a live feed matrix allows for sustained, non-disruptive oral administration that perfectly aligns with existing hatchery rearing protocols.

The development of stable and heritable expression systems is therefore a critical prerequisite for advancing rotifer-based RNAi applications. While the CRISPR–Cas9 system was recently adapted for rotifers using electroporation, these early attempts primarily affected somatic tissues. The resulting mutations were largely mosaic and lacked stable germline transmission (Kim et al., 2019). Transgenic technologies provide a promising solution to achieve durable expression of antiviral RNA molecules in live feed organisms. Among available genetic tools, the piggyBac transposon system has been widely applied for stable genomic integration and long-term transgene expression in diverse invertebrate species (Lobo et al., 2006). Owing to its high integration efficiency and genetic stability, piggyBac represents an attractive platform for developing transgenic systems in non-model marine organisms. However, the potential of piggyBac-mediated transgenesis in marine rotifers, particularly for antiviral RNAi applications in aquaculture, has not been systematically explored.

In this study, we establish a transgenic rotifer-based RNA interference strategy aimed at antiviral protection against CMNV in shrimp aquaculture. By combining stable shRNA expression in *B. plicatilis* with its established role as live feed for *P. vannamei*, this work proposes an application-oriented framework for RNAi-mediated immunoprophylaxis that is compatible with existing shrimp farming practices. Rather than altering current culture systems, this strategy leverages an already indispensable component of shrimp hatcheries to deliver antiviral siRNA in a sustainable manner. Our findings provide a proof of concept for the functional use of genetically engineered live feed organisms and highlight their potential as a novel tool for viral disease management in shrimp aquaculture.

## 2 Results

### 2.1 Comprehensive quality assessment and validation of a high-fidelity *B. plicatilis* reference genome

**Figure 1:**
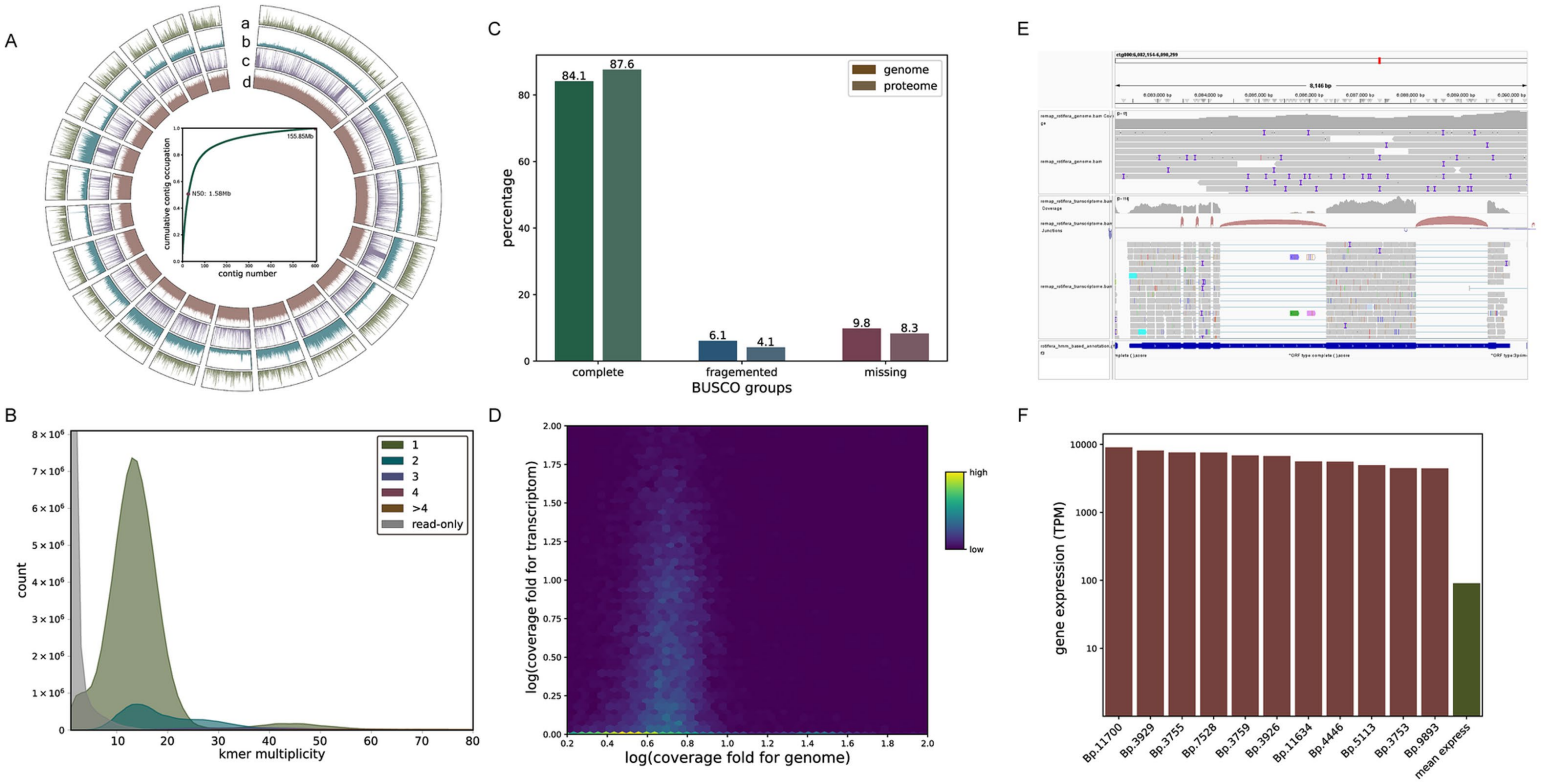
Genomic profiling for *Brachionus plicatilis*. **(A)** Circos for the top 20 longest contig of *B. plicatilis* genome. Tracks from outer to inner are (a) gene density, (b) genome coverage, (c) transcriptome coverage, and (d) GC content. Cumulative ratio of contig length relative to the total genome size is plotted in the center. **(B)** Merqury *k*-mer spectra-copy number analysis of the *B. plicatilis* genome assembly. **(C)** BUSCO completeness assessment of the *B. plicatilis* genome and predicted proteome. **(D)** Hexbin plot of log-scaled transcriptomic coverage versus log-scaled genomic coverage across 10-kb sliding windows. **(E)** IGV track showing genome coverage (upper panel) and transcriptome coverage (lower panel) for the gene. **(F)** Relative gene expression level for top 11 genes (dark red) compared to average gene expression of *B. plicatilis* (dark green).

To facilitate stable genetic manipulation in *B. plicatilis*, comprehensive genomic and transcriptomic resources were generated and systematically evaluated. Isolating pure genomic material from live rotifers is notoriously challenging due to their massive ingestion of microalgae, which severely contaminates sequencing libraries and can lead to misassembled chimeric contigs. To bypass this biological hurdle, adult rotifers were subjected to a rigorous starvation treatment prior to nucleic acid extraction, successfully inducing the clearance of algal contents from their digestive tracts and minimizing exogenous algal DNA contamination. *De novo* genome assembly yielded a highly accurate genome draft consisting of 607 contigs with a contig N50 of 1.58 Mb and a total assembly size of 155.85 Mb (Figure 1A). To evaluate the base-level accuracy and structural reliability of the assembly, we performed a *k*-mer spectra analysis using merqury. The evaluation revealed an exceptional consensus quality score of QV 56.30 and a clear single-copy peak matching the expected haploid coverage, with negligible redundancy and collapsed repeats (Figure 1B). Furthermore, BUSCO assessment employed against the metazoa_odb10 database demonstrated high gene-level completeness, capturing 84.1% of core conserved genes as complete at genome-level metric, which further improved to 87.6% while evaluating using predicted proteome (Figure 1C). Considering the difficulty in obtaining adequately pure material of *B. plicatilis*, these metrics confirmed that the assembly possesses the high continuity and single-base resolution required to reliably support downstream gene annotation and regulatory element discovery.

To assess the macro-scale consistency of the assembled genome, both genomic DNA and transcriptomic sequencing reads were mapped back to the contigs. The genomic and transcriptomic coverages were calculated with 10 k sliding windows. The transcriptomic coverage across sliding windows primarily centered around 2.1-fold of the genomic depth, demonstrating a highly consistent and uniform distribution across different genomic regions, providing the sensitivity required to accurately capture transcriptomic variations (Figure 1D). Visualization of representative loci revealed well-defined exon–intron structures and consistent RNA-seq coverage across expressed genes, further supporting the integrity of the genome assembly (Figure 1E).

Based on this validated genome-transcriptome foundation, transcriptional profiling was performed to identify endogenously highly expressed genes in *B. plicatilis.* Expression analysis revealed a subset of genes exhibiting robust and stable expression, which were selected as candidates for promoter mining (Figure 1F).

Taken together, these comprehensive evaluations—spanning base-level precision, single-copy haplotype resolution (Figure 1B), and core gene-level completeness—demonstrate that this assembly provides a highly accurate and stable reference genome, establishing a solid foundation for subsequent high-resolution gene feature identification and functional genetic tool development in *B. plicatilis.*

### 2.2 Stable expression and germline transmission of a reporter gene in transgenic rotifers

Leveraging the integrated genomic and transcriptomic datasets, we identified *Bp.3757* as a core, highly expressed gene candidates through our bioinformatic pipeline. To utilize this element for functional validation, a 2-kb regulatory region immediately upstream of the *Bp.3757* translation initiation codon (ATG) at coordinate ctg004:1,703,733 was cloned and designated as *pBp3757* (referred to herein as the “promoter”). Driven by this endogenous promoter, both the donor and helper constructs were designed to enable stable genomic integration and transposase expression in rotifers. This strategy effectively lays the foundation for establishing a functional, piggyBac-mediated transgenic platform for *B. plicatilis*.

**Figure 2:**
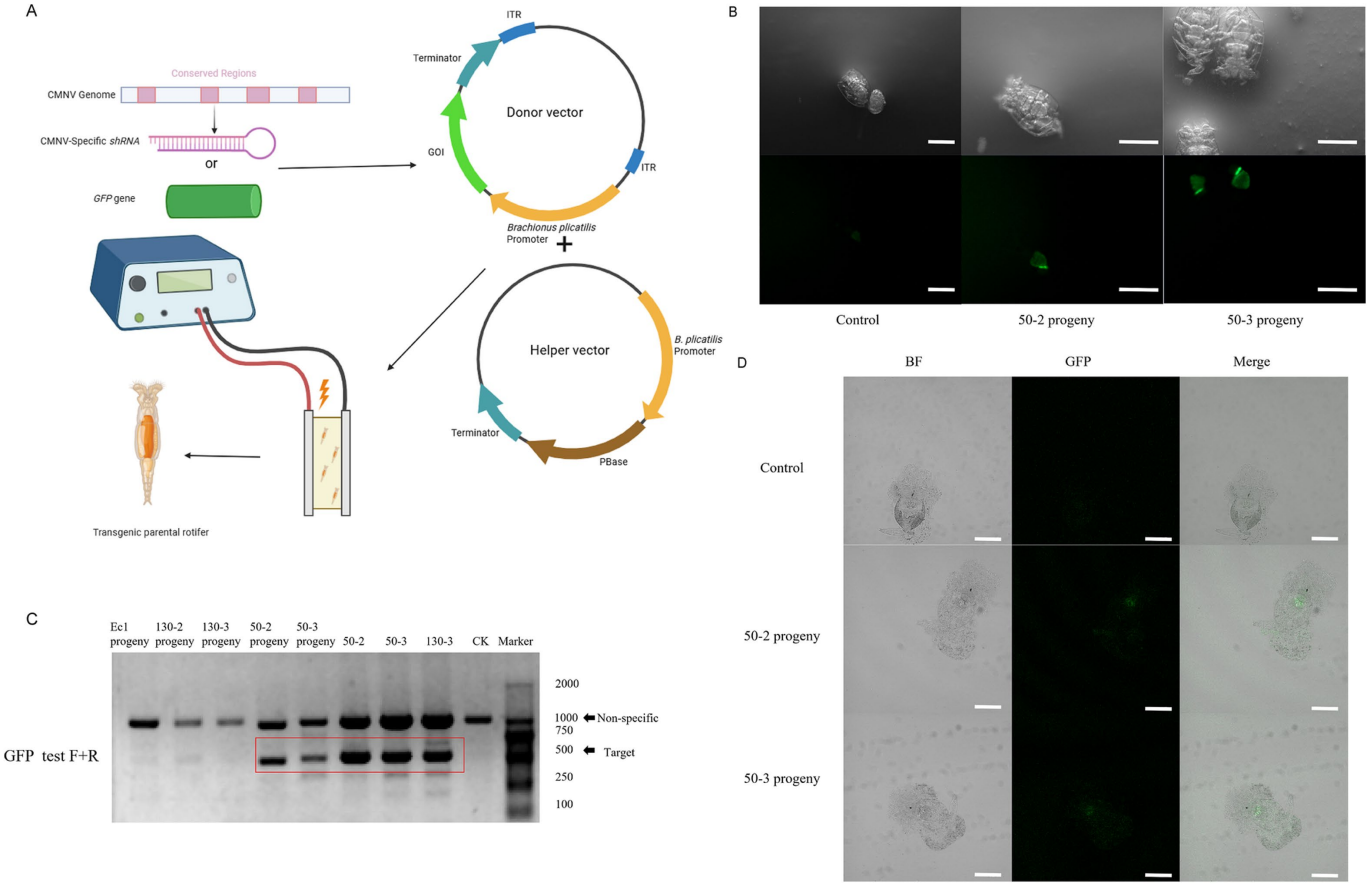
Design and validation of the piggyBac-mediated transgenic platform in *B. plicatilis*. **(A)** Schematic diagram of the dual-plasmid piggyBac system. Donor vector: contains the GOI (Gene of Interest, e.g., *GFP* or *shRNA*) driven by an endogenous *B. plicatilis*. Promoter and followed by a Terminator, all flanked by ITR (Inverted Terminal Repeats) for genomic integration. Helper vector: contains the *PBase* (piggyBac Transposase) gene. **(B)** Bright-field (BF) and GFP fluorescence images of rotifers from the control group and the electroporation group. Bar scale = 100 μm. **(C)** PCR verification of heritable *GFP* integration. The 540 bp band corresponds to the target *GFP* sequence, while the ~1,000 bp band is a non-specific amplification byproduct. Lanes labeled with numeric codes (e.g., 50–2, 130–3) indicate pulse duration (ms) and pulse frequency. Ec1 indicates a preset *Escherichia coli* electroporation program used as a reference. “Progeny” indicates offspring sampled 30 days after the initial treatment. **(D)** Laser confocal scanning microscopy images of electroporated rotifers showing BF, GFP, and merged channels. Bar scale, 100 μm.

To evaluate the heritability of the transgene, PCR was performed on GFP-positive parent rotifers under a macro zoom stereo microscope and their progeny sampled 30 days post-electroporation (Figure 2B). As shown in Figure 2C, the target *GFP* fragment (540 bp) was consistently detected across various treatment conditions (e.g., 50–2, representing a 50 ms pulse duration with 2 pulses). Ec1, a preset program for bacterial transformation, was also included for comparison. While a higher-molecular-weight non-specific band (~1,000 bp) was observed in all samples, including the control, the specific 540 bp band remained present in the offspring samples of groups 50–2 and 50–3 after multiple generations of culture; this confirms that the construct had stably integrated into the genome and achieved germline transmission.

We next examined *GFP* expression by laser confocal microscopy (Figure 2D). For high-resolution sub-cellular localization of GFP, rotifers were physically immobilized to prevent motion blur during confocal Z-stack acquisition. While the requisite imaging conditions (e.g., prolonged laser exposure) resulted in localized salinity stress, the specificity of the GFP signal was confirmed by comparing these results with wild-type controls treated under identical conditions. Clear green fluorescence was consistently observed in the transgenic lines, predominantly localized in metabolic tissues, whereas no signal was detected in the control group.

### 2.3 Identification of CMNV in farmed *P. vannamei* and generation of transgenic rotifers expressing CMNV-specific shRNA

**Figure 3:**
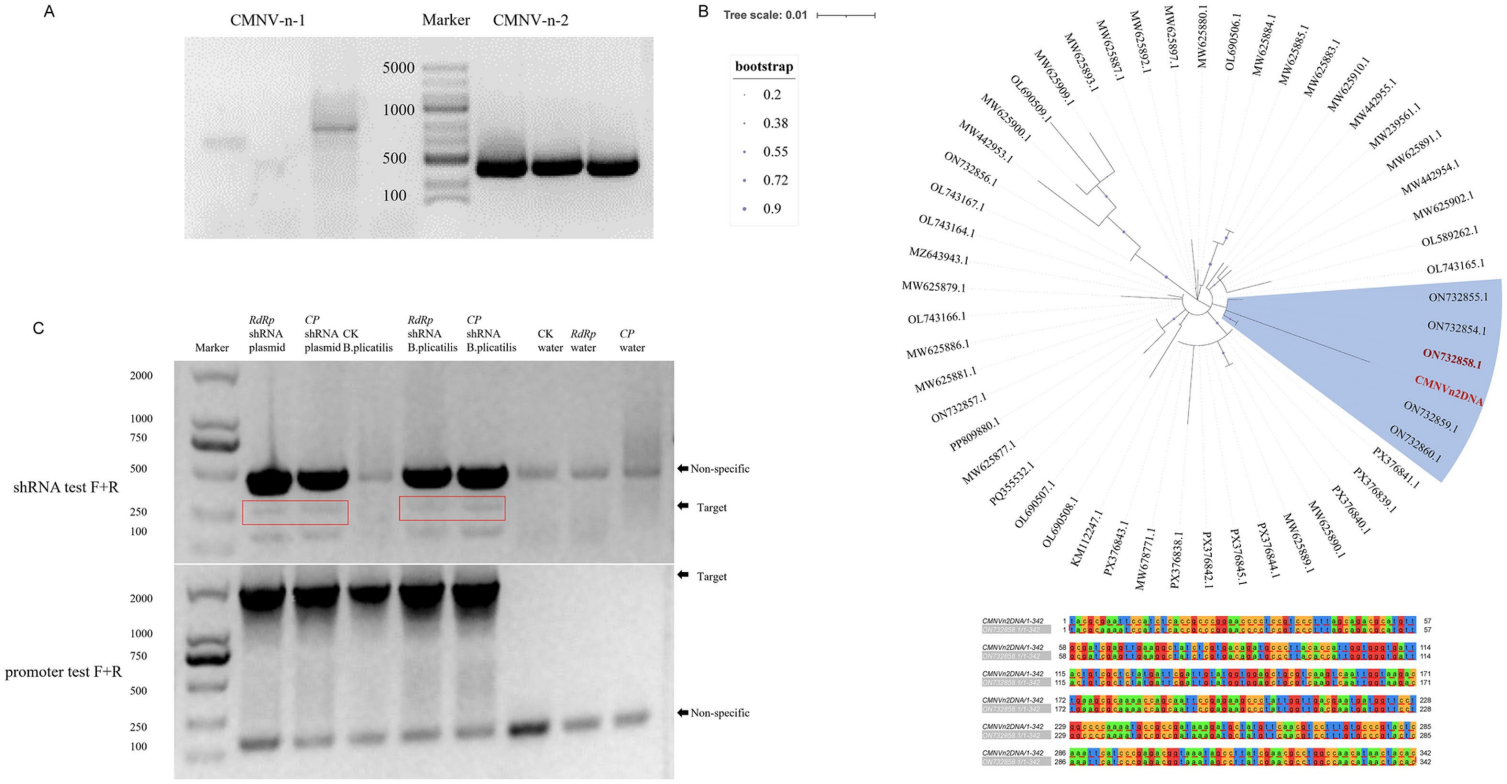
**(A)** RT-Nested PCR detection of the CMNV *RdRp* fragment from VCMD samples. **(B)** Upper panel: Phylogenetic tree of different CMNV isolates and other *Nodaviruses* based on *RNA dependent RNA polymerase* (*RdRp*) gene generated by the neighbor-joining method using the MEGA X. Lower panel: The nucleotide sequences of CMNVn2DNA and ON732858.1 were aligned and visualized with color-coded bases. **(C)** PCR verification of *CMNV-shRNA* cassette integration in transgenic rotifers. The upper panel shows the shRNA test (target band at 246 bp); the lower panel shows the endogenous promoter control (2,000 bp).

Although the appearance of non-specific bands is expected in the first round of nested PCR due to the high sensitivity of the primers (Zhang et al., 2014), all secondary PCR products in this study yielded single, distinct bands of the expected 413 bp targeted gene amplicons in all three samples (Figure 3A). The sequences of the PCR products were subjected to BLAST analysis. BLAST analyses indicated that the obtained sequence exhibited high sequence similarity with CMNV *RdRp* sequences, with the most similar matches being ON732858.1 (95% query coverage, 99.42% identity), This isolate also originated from VCMD samples from the Yellow Sea region of China (Yao et al., 2022). The phylogenetic analysis showed that CMNVn2DNA was located within the phylogenetic region containing known CMNV-related *RdRp* sequences,including ON732858.1 (Figure 3B). Based on this result, this CMNV isolate strain was selected as the target virus for subsequent RNA interference-based control.

To enable targeted antiviral gene silencing, conserved regions of the CMNV genome were selected for the design of shRNA constructs. The CMNV-specific shRNA expression cassette was incorporated into the established piggyBac donor vector under the control of endogenous *B. plicatilis* promoter elements, generating a transgenic construct suitable for stable expression in rotifers (Figure 2A).

Using the piggyBac-mediated transgenic platform developed in this study, rotifers expressing shRNA targeting the CMNV *RNA-dependent RNA Polymerase* (*RdRp*) and *coat protein* (*cp*) gene were successfully obtained. PCR analysis of genomic DNA confirmed the presence of the *CMNV-shRNA* cassette in transgenic rotifers, indicating stable genomic integration of the antiviral construct, Faint but distinct bands at 246 bp confirm the presence of the shRNA cassette; larger bands represent non-specific amplification common with structured templates (Figure 3C). It should be noted that while genomic integration was confirmed, the specific transcription levels of the shRNA and its subsequent processing into mature siRNA within *B. plicatilis* were not directly quantified in this study. The generation of *CMNV-shRNA*–expressing rotifers established a direct link between pathogen identification in shrimp and the development of a functional, feeding-compatible RNA interference tool, enabling subsequent evaluation of antiviral efficacy in *P. vannamei*.

### 2.4 Feeding transgenic rotifers confers antiviral protection against CMNV in *P. vannamei* under aquaculture-relevant conditions

**Figure 4:**
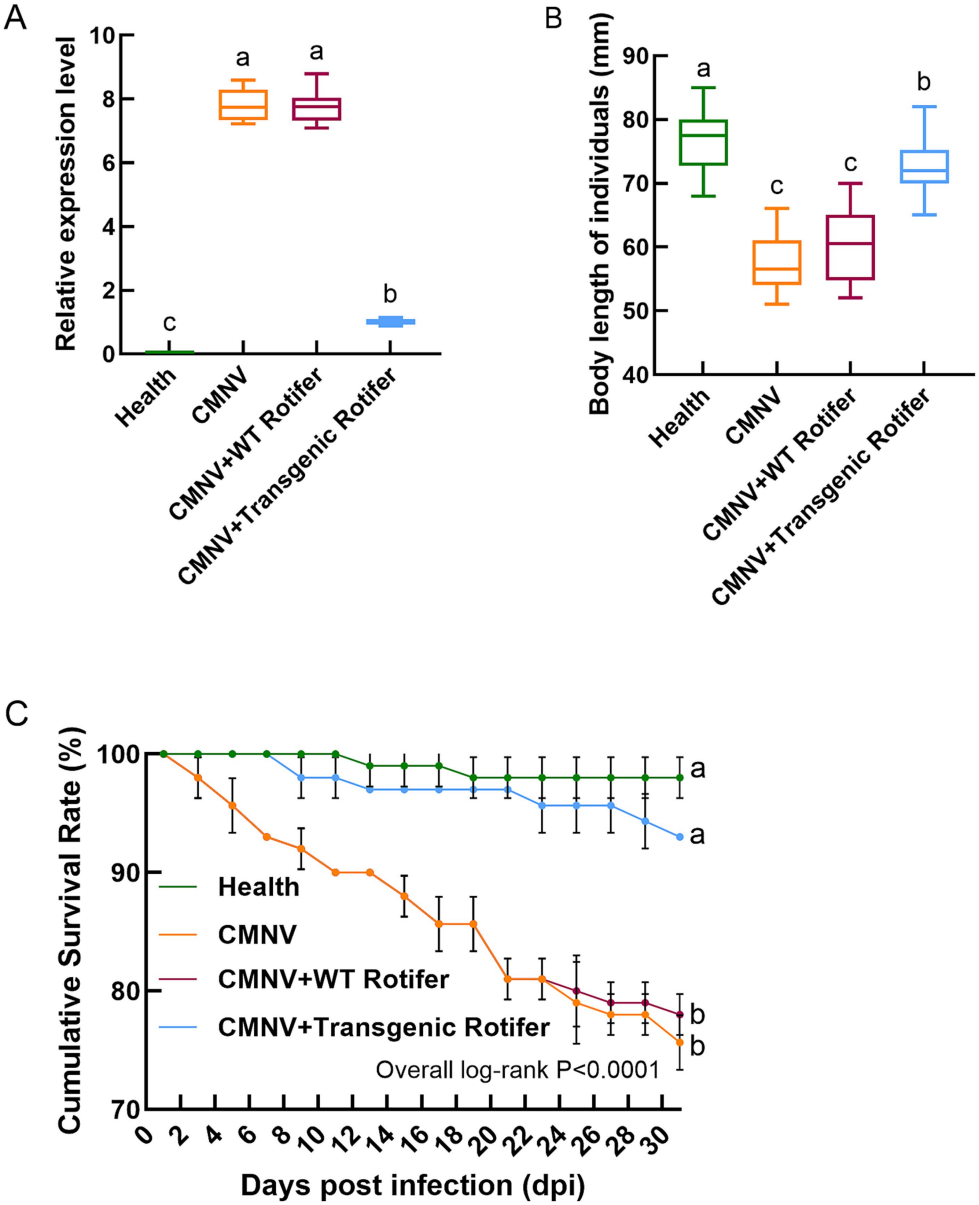
Effects of feeding *CMNV-shRNA*–expressing rotifers on CMNV infection in *Penaeus vannamei*. **(A)** Relative expression levels of CMNV in shrimp from different treatment groups. **(B)** Body length of shrimp at the end of the experiment. **(C)** Cumulative survival rate of shrimp over 31 days post CMNV infection. Health, healthy control group; CMNV, CMNV-infected group without rotifer treatment; CMNV + WT Rotifer: CMNV-infected group fed non-transgenic rotifers; CMNV + Transgenic Rotifer: CMNV-infected group fed *CMNV-shRNA*–expressing transgenic rotifers. For **(A)** and **(B)**, box plots show the median, interquartile range, and minimum-to-maximum values. Statistical differences in **(A)** and **(B)** were determined by one-way ANOVA followed by Tukey’s multiple-comparisons test. Survival curves in **(C)** are shown as means ± SE from three replicate tanks, with 30 shrimp per replicate, and were compared using the log-rank test. Different letters indicate significant differences among groups (*p* < 0.05); groups sharing the same letter are not significantly different.

To evaluate the antiviral efficacy of *CMNV-shRNA*-expressing rotifers, *P. vannamei* were fed transgenic rotifers under controlled laboratory conditions, and viral infection levels were quantified. We utilized a screened transgenic population (verified via PCR in Figure 3C) rather than transient F0 individuals. Quantitative PCR results revealed a significant effect of treatment on relative CMNV expression level in shrimp fed *CMNV-shRNA*-expressing rotifers was significantly lower than that in the CMNV + wild-type (WT) Rotifer group (Figure 4A). The relative viral expression level in the CMNV + Transgenic Rotifer group was reduced by approximately 87.1% compared with the CMNV + WT Rotifer group. In the healthy control group, the CMNV target was not detected. These results show that feeding transgenic rotifers strongly reduced CMNV replication in shrimp.

We further assessed the protective effect of transgenic rotifers by measuring shrimp growth performance under infection pressure. Feeding transgenic rotifers had no adverse effects on shrimp body length. The body length of shrimp in the CMNV + Transgenic Rotifer group reached 72.77 ± 0.80 mm at the end of the experiment, which was significantly longer than those in the CMNV group (57.47 ± 0.77 mm) and the CMNV + WT Rotifer group (60.10 ± 0.98 mm) (Figure 4B). This value was also comparable to that in the healthy control group (76.77 ± 0.82 mm). Thus, shrimp fed *CMNV-shRNA*-expressing rotifers maintained normal growth performance under CMNV infection.

Survival was also monitored during the infection experiment (Figure 4C). Mortality started around 4–6 days post-infection (dpi) in the CMNV-infected groups. At 31 dpi, cumulative survival in the CMNV group decreased to about 75.67 ± 1.33%. The CMNV + WT rotifer group showed a similar survival of about 78.00 ± 1.00%. In contrast, shrimp fed *CMNV-shRNA*-expressing rotifers maintained a higher survival of about 93.00 ± 0.00%, which was significantly higher than both CMNV-infected control groups based on pairwise log-rank tests with Bonferroni correction.

In summary, these results indicate that feeding *CMNV-shRNA*-expressing transgenic rotifers reduces the CMNV viral load, promotes shrimp growth, and enhances survival rates under CMNV infection—findings consistent with the protective effects mediated by CMNV-targeted RNAi. This demonstrates that this feeding strategy provides a distinct protective effect against CMNV in *P. vannamei*.

## 3 Materials and methods

### 3.1 Rotifer and shrimp culture

The *B. plicatilis* strain was cultivated in artificial seawater (ASW) with a salinity of 30 parts per thousand (ppt), prepared using concentrated sea salt (Blue Grand Star, China). The clone strain was maintained in an illuminated incubator set to a light intensity of 4,000 l×, with a 12-h light: 12-h dark photoperiod, at a temperature of 25 °C. The culture method for the rotifers was based on Snell’s methods with minor adjustments (Snell et al., 2011). The animal rights are not applicable.

The rotifers were fed once daily with a concentrated suspension of *Chlorella vulgaris* at a density of 3 × 10^6^ cells/mL (Zhang et al., 2024). The algae *C. vulgaris* were cultured in F/2 medium at 25 °C, under continuous light exposure of 4,000 l×. The algae *C. vulgaris* were harvested at the exponential growth phase using centrifugation at 5,000 rpm for 10 min.

Specific pathogen-free (SPF) juvenile *P. vannamei* were kept in glass aquaria (20 L) with ASW (30 ppt) and a sterile uniform gravel bed. The water temperature was maintained at 25 °C. A weekly 30% water change through siphoning. Feeding was performed with commercial pellets three times a day at 3% of the bodyweight (biomass).

### 3.2 *De novo* genome assembly and comprehensive quality evaluation

To construct a high-quality reference genome for *B. plicatilis*, clean PacBio HiFi long reads were subjected to *de novo* genome assembly. Sequencing data quality and read length distribution were initially verified using NanoPlot (v1.46.2). The *de novo* assembly was executed using Hifiasm (0.25.0-r726) with parameters (−l0 --primary) tailored for high-fidelity long reads, generating the primary contig graphs. No scaffolding with gap-filling was performed, ensuring that the final assembly maintained true physical continuity.

To guarantee the reliability and reproducibility of the assembly, rigorous multi-dimensional evaluations were implemented. Base-level consensus accuracy, error rates, and haplotype completeness were quantified using Merqury (v1.3) with a standardized *k*-mer size of *k* = 21, benchmarked directly against the un-assembled HiFi reads. Biological and gene-level completeness was systematically assessed using BUSCO (v5.8.0) against the metazoa_odb10 lineage dataset in both genomic (−m genome) and protein (−m prot) modes. The basic assembly statistics, including contig N50, total assembly size, and contig lengths, were calculated and extracted via standard bioinformatic operations.

### 3.3 Transcriptome alignment and gene expression quantification

The raw RNA-seq reads were subjected to quality control, genomic mapping, and expression quantification. High-quality clean reads were obtained using fastp (v1.0.1) with default filtering parameters to remove low-quality bases, adapter sequences, and poly-N tails.

The filtered transcriptomic reads were subsequently mapped against the newly assembled *B. plicatilis* contigs using HISAT2 (v2.2.1) with splice-aware alignment parameters (−-dta). Alignment metrics and overall mapping rates were extracted using SAMtools (v1.20). A stringent, multi-step annotation workflow was implemented to define trustworthy protein-coding gene models. Initial transcript models were assembled using StringTie (v3.0.0). To eliminate transcriptional noise and non-coding structural artifacts, potential open reading frames (ORFs) were systematically scanned and filtered using HMMER (v3.4) alongwith P-fam models by default parameters. Only high-confidence transcripts successfully validated by HMM thresholds were retained. These refined locus architectures were subsequently integrated to generate a definitive, non-redundant reference annotation file (GFF3), from which corresponding proteome sequences were extracted.

To calculate gene expression abundance, fragment counts per gene locus were quantified using feature Counts (v2.0.8) based on the structural GFF3 annotations. Transcriptional abundance was normalized into Transcripts Per Million (TPM) values to rule out gene length and sequencing depth biases. Loci displaying robust and high expression were mathematically prioritized as core candidates for subsequent upstream promoter dissection.

### 3.4 Extraction of total RNA, DNA and cDNA synthesis

Rotifer DNA was extracted using the Hotshot method (Fontaneto et al., 2015). The alkaline lysis buffer contained 25 mmol/L NaOH and 0.2 mmol/L EDTA (pH 8.0), and the neutralization buffer contained 40 mmol/L Tris–HCl (pH 5.0). Samples were lysed in 10 μL alkaline lysis buffer in PCR tubes at 95 °C for 30 min, frozen at −20 °C for 5–10 min, and then mixed with 10 μL neutralization buffer. DNA samples were stored at −20 °C until use.

The total RNA of *P. vannamei* was prepared using an RNAprep pure Tissues Kit (Tiangen, China) according to the manufacturer’s protocol. The purity and concentration of the extracted RNA were measured by Nanodrop 2000 (Thermo Scientific, USA), and then RNA reverse transcription into cDNA using ABScript Neo RT Master Mix for qPCR with gDNA Remover (ABclonal, China).

### 3.5 Construction of piggyBac-based transgenic vectors

A piggyBac transposon system was used to establish stable transgenesis in *B. plicatilis* (Figure 2A). The system consisted of two plasmids: a donor plasmid carrying the transgene expression cassette flanked by piggyBac inverted terminal repeats regions, and a helper plasmid encoding codon-optimized hyperactive piggyBac transposase under the control of the selected endogenous *B. plicatilis* promoter, the transposase gene was synthesized by CWBIO Co., Ltd. (China).

Endogenous rotifer promoter sequences identified from genome and transcriptome analysis were cloned upstream of reporter genes or shRNA expression cassettes to replace heterologous viral promoters. For reporter validation, a GFP expression cassette was constructed. For antiviral applications, *CMNV-shRNA* expression cassettes were cloned downstream of the selected endogenous promoter and 5′ UTR, followed by the SV40 poly(A) signal.

All target gene fragments and vector backbones were amplified using KOD One PCR Master Mix (TOYOBO, Japan) and assembled using the pEASY^®^-Basic Seamless Cloning and Assembly Kit (TransGen Biotech, China). The inserted fragments and junction regions were verified by Sanger sequencing before use in rotifer electroporation.

### 3.6 *CMNV-shRNA* design

The CMNV sequence obtained in this study was aligned with representative reported CMNV sequences to identify conserved regions suitable for RNAi targeting. Conserved regions within the *RNA-dependent RNA polymerase* (*RdRp*) gene and the *coat protein* (*cp*) gene were selected as candidate regions for shRNA design. Candidate sequences were submitted to the Thermo Fisher Scientific BLOCK-iT™ RNAi Designer, with *B. plicatilis* selected as the reference organism to reduce potential off-target risk against rotifer endogenous sequences. Based on the recommended results from this tool and the conservation of the target sites among different CMNV sequences, one 21-nt target site was selected from each of the *RdRp* and *cp* genes.

Each shRNA expression cassette was designed in a sense strand–loop sequence–antisense strand format and cloned into the piggyBac donor plasmid downstream of the selected endogenous *B. plicatilis* promoter and 5′ UTR. The detailed shRNA target positions, target sequences, sense strands, loop sequences, antisense strands, and relevant cloning/flanking sequences are listed in Supplementary Table S1.

### 3.7 Electroporation

To electroporate exogenous macromolecules into adult *B. plicatilis* (approximately 30 individuals per replicate), a Gene Pulser Xcell™ Electroporation System (Bio-Rad, USA) was used. Prior to electroporation, rotifers were gently washed and suspended in low-salinity medium (1 psu) to reduce ionic interference and improve electroporation efficiency while maintaining organismal viability.

Electroporation was performed under the following conditions: a voltage of 250 V, capacitance of 50 μF, resistance of 1,000 *Ω*, and a pulse duration of 50 ms. Each sample received 2–3 square-wave pulses with brief intervals between pulses. These parameters were selected based on preliminary optimization trials, in which this configuration yielded a post-electroporation survival rate of approximately 20–30%, significantly outperforming protocols with longer pulse durations (e.g., 130 ms) which resulted in higher mortality (80–90%). Immediately after electroporation, rotifers were transferred to seawater with a salinity of 15 psu for recovery and subsequent culture.

After a 24-h recovery period post-electroporation, individual rotifers were isolated and transferred into 96-well plates (one individual per well) to establish clonal cultures. Clonal lines exhibiting GFP fluorescence were identified using a macro-zoom stereo fluorescence microscope and subsequently expanded. To ensure stable genomic integration and eliminate potential false positives from residual plasmids, the identified lines were cultured for at least 30 days (equivalent to multiple generations). During this period, rotifers were periodically collected using a cell strainer (40 μm mesh) and transferred to fresh artificial seawater to dilute and remove extracellular DNA. Only lines showing persistent, heritable fluorescence after 30 days were considered successfully integrated and used for further experiments.

### 3.8 Fluorescence microscopy and confocal imaging

Prior to imaging, rotifers were starved for 24 h to clear the digestive tract. Samples were collected using a 40 μm cell strainer, washed three times with filtered artificial seawater, and mounted in a minimal volume to restrict swimming for stable confocal imaging. The specific GFP fluorescence and tissue localization in transgenic *B. plicatilis* were initially characterized using a macro zoom stereo microscope (ZEISS, Germany). For high-resolution subcellular localization, live rotifers were mounted on glass slides and imaged using a laser confocal microscope (Leica, Germany). The excitation and emission wavelengths were 473 and 520 nm for GFP. Image acquisition and processing were performed using the Leica Application Suite X (LAS X) software.

### 3.9 Virus purification

A total of 3 samples of *P. vannamei* (about 3–8 g in body weight) obtained from 2 local farms in Qingdao City, China, in December 2025. The cephalothoraxes were dissected aseptically and homogenized in sterile PBS using a mortar and pestle or preserved immediately in RNA store solution (Tiangen Biotech, China). The homogenate liquid was centrifuged at 10,000 g for 25 min at 4 °C to remove tissue debris, and the supernatant was filtered through a 0.22 μm membrane and then centrifuged at 130,000 g for 4 h. The precipitation was resuspended as viral inoculum for the challenge test.

### 3.10 Determine the evolutionary relationship of CMNV by RT-nPCR and phylogenetic tree analysis

VCMD samples were amplified for sequencing using a two-step reverse transcription nested PCR (RT-nPCR) assay with two pairs of primers. Briefly, a 619-bp fragment was obtained in the first round, followed by a second-round PCR that generated a 413-bp target amplicon. All amplification reactions were performed using 2 × Rapid Taq Master Mix (Vazyme, China). The primer sets and amplification procedures were the same as those described previously (Wang et al., 2021b). Following amplifications, products were separated in an agarose gel electrophoresis and bands were sequence verified at Sangon Biotech Co., Ltd. (China).

The sequence was identified through BLAST searches, and the deduced amino acid sequences of CMNV target *RdRp* gene fragments from positive samples and *RdRp* amino acid sequences from other nodavirus were selected for phylogenetic analysis by using MEGA X software (Kumar et al., 2018).

### 3.11 Challenge test

Transgenic rotifers expressing CMNV-specific shRNA were mass-cultured and used as live feed for Shrimp were divided into experimental groups receiving either shRNA-expressing rotifers or non-transgenic control rotifers. It should be noted that the challenge experiments were conducted using stable transgenic lines—specifically, lines that had been maintained for at least 30 days following electroporation—to ensure genomic stability.

For antiviral efficacy assessment, shrimp were subjected to an indoor CMNV challenge experiment. Viral exposure was conducted under controlled laboratory conditions. Each group included three replicates and each replicate included 30 individuals. Then, 5 μL of viral inoculum (about 5.5 × 10^6·5^ copies/μL) (or TN buffer) were injected intramuscularly into the third abdominal segment of each healthy shrimp individual to be serving as the infection group. Thereafter, the shrimp were maintained with rotifers or pellet feeds for 31 days in tanks, with mortality rates recorded daily, and cumulative survival rate were generated. At the end of the challenge period, shrimp were sampled for molecular analysis and growth assessment.

Total RNA was extracted from shrimp tissues collected at defined time points post-challenge. Quantitative real-time PCR (qRT-PCR) was performed to quantify CMNV RNA levels by using Hieff UNICON^®^ Advanced qPCR SYBR Master Mix (Yeasen, China) using shrimp *glyceraldehyde-3-phosphate-dehydrogenase* (*gapdh*) gene as reference gene (Valenzuela-Castillo et al., 2017). Relative viral loads were calculated using the 2^−ΔΔCt^ method.

In parallel, shrimp body length and body weight were measured at the end of the experiment. Weight gain and specific growth rate were calculated to evaluate potential effects of transgenic rotifer feeding on shrimp growth performance. All primer sequences in this study are listed in Supplementary Table S1.

### 3.12 Statistical analysis

All statistical analyses were performed using GraphPad Prism version 8.0. For comparisons involving two groups, Student’s *t*-test was used. For comparisons among the four treatment groups in Figures 4A,B, data were analyzed using one-way ANOVA followed by Tukey’s multiple-comparisons test. Survival curves in Figure 4C were compared using the log-rank (Mantel-Cox) test, and pairwise comparisons were adjusted using the Bonferroni method. Data are presented as means ± standard error (SE) unless otherwise stated. A *p-*value < 0.05 was considered statistically significant.

## 4 Discussion

The lipid-rich and highly prolific rotifer *B. plicatilis* serves as an suitable model in aquatic ecology, ecotoxicology, and live feed aquaculture (Kim et al., 2018). However, the lack of continuous and structurally validated reference genomes has long hindered high-throughput functional genomics and precise genetic engineering in this phylum. In this study, by meticulously starvation treatment to bypass microalgal contamination, we generated a high-fidelity assembly characterized by a Contig N50 of 1.58 Mb and an exceptional single-base accuracy (QV 56.30). Beyond serving as a methodological platform for mining the endogenous promoters utilized in our *piggyBac* transgenic system, this extensive genomic and transcriptomic dataset holds broader biological significance for the scientific community. It provides a clean, contamination-free genomic blueprint that can accelerate the systematic identification of gene families involved in stress tolerance, reproductive switch mechanisms, and xenobiotic detoxification in monogonont rotifers.

Viral diseases remain a major challenge in shrimp aquaculture and often cause large economic losses (Richard et al., 2023). In contrast to the natural environment, shrimp culture in farms with high densities and unstable water conditions can easily form outbreaks once virus infection occurs, making it difficult to control (Liao et al., 2022). In addition, shrimp only have innate immunity, a type of non-specific immune response made up mostly of cellular defense and humoral defense (Wang and Zhang, 2008), which limits the use of classical vaccination strategies.

RNA interference (RNAi) has been widely studied as an antiviral mechanism in Crustacean Aquaculture (Fajardo et al., 2024). Previous studies have shown that RNA molecules targeting viral genes can suppress viral replication in shrimp (Zhong et al., 2026; Alam et al., 2023). Although RNAi technology has shown strong antiviral effects in shrimp, its practical use in aquaculture remains limited (Kitagishi et al., 2011). Many previous studies delivered dsRNA through injection (Tirasophon et al., 2005; Yodmuang et al., 2006; Attasart et al., 2010). Therefore, one of the challenges in successfully translating RNAi therapy from the laboratory to practical application is how to deliver RNA molecules into living organisms (Alam et al., 2023). The administration of RNAi therapeutic molecules through oral delivery has shown success in various arthropod species. This can be achieved by delivering the molecules in a naked form, conjugated with a polymer, or using bacteria that carry specific dsRNA/siRNA. Additionally, incorporating the molecules into the feed of fish or shellfish has also been effective (Attasart et al., 2013; Treerattrakool et al., 2013; Thammasorn et al., 2013). It can be said that the oral route is currently the most promising and cost-effective method for RNAi delivery in aquatic environments.

Rotifers are widely used as live feed in shrimp hatcheries because they reproduce rapidly and can be cultured at large scale (Hagiwara et al., 2007). These characteristics make them suitable carriers for functional molecules. This is why rotifers were used as carriers for antiviral RNA in this study. We developed a feeding-based RNAi strategy using transgenic *B. plicatilis*. Using a piggyBac-mediated transgenic system, we obtained rotifers with stable genomic integration and heritable expression of CMNV-specific shRNA. Shrimp fed these rotifers showed lower viral load and higher survival during CMNV infection. In addition, feeding transgenic rotifers did not reduce shrimp growth, suggesting that this strategy can be incorporated into routine culture conditions. To our knowledge, few studies have examined the use of transgenic rotifers for RNAi-based antiviral delivery in shrimp aquaculture. It is worth noting that highly efficient, heritable gene editing in rotifers has been achieved through microinjection. However, the precision of that method requires intensive manual labor, making it difficult to scale up. In contrast, our piggyBac-mediated platform offers a more practical and scalable alternative for mass-producing the engineered populations required for commercial shrimp farming (Feng et al., 2023). The urgent need for effective CMNV control is further underscored by its recently discovered capacity for zoonotic transmission to humans via unprotected contact with aquatic animals. By significantly reducing the viral load in farmed shrimp by approximately 90%, our transgenic rotifer-based RNAi platform not only protects the aquaculture industry but also functions as a critical biosecurity barrier. Reducing the prevalence of CMNV at the production stage could substantially mitigate the risk of human exposure during downstream processing and consumption, thereby addressing an emerging public health challenge (Liu et al., 2026).

While this proof-of-concept study demonstrates the platform’s viability, several methodological limitations remain to be addressed. First, the distinct localization of the GFP signal within the digestive and metabolic tissues warrants deeper investigation. Given our strict 24-h starvation and washing protocols, this pattern is highly unlikely to arise from algal food residues; rather, it probably reflects the intrinsic spatial activity of the selected endogenous promoter or localized differences in cellular metabolic rates. To definitively map this expression profile across all somatic tissues, future efforts will require the deployment of ubiquitously expressed housekeeping promoters.

Beyond the reporter system, establishing the molecular precision of the antiviral platform introduces additional priorities. For instance, although wild-type rotifers served as a primary control for the feeding treatment, this study did not include a non-targeting shRNA transgenic rotifer control. The inclusion of such a control in future work would be ideal to more definitively confirm the sequence-specific nature of the observed antiviral RNAi effects. Another important consideration for future research is the detailed characterization of the RNAi machinery within the transgenic rotifers. Although our feeding experiments demonstrated clear antiviral efficacy in *P. vannamei*, the intermediate steps—specifically the transcription of shRNA and its enzymatic processing into mature siRNA by the rotifer’s endogenous Dicer—remain to be empirically verified through small RNA sequencing or Northern blot analysis. Ultimately, translating these molecular insights into practical aquaculture applications will require shifting from controlled experimental models to farm-scale realities. While intramuscular injection in this proof-of-concept study provided a standardized challenge for the initial assessment of efficacy, field or farm-scale trials are still required; validation using oral administration or natural infection routes is crucial for further evaluating the practical utility of this platform.

In parallel with these technical advancements, environmental biosafety and biocontainment strategies are paramount when introducing transgenic live feeds into aquaculture ecosystems. The transgenic *B. plicatilis* platform developed in this study must be strictly utilized within fully contained, closed-recirculation indoor facilities. To mitigate the risk of accidental escape into local aquatic ecosystems, a multi-barrier containment strategy must be implemented. All hatchery effluent should pass through consecutive physical barriers, including micro-mesh filters capable of retaining rotifers and their resting eggs. Subsequently, downstream wastewater must undergo reliable sterilization procedures—such as high-dose ultraviolet (UV) irradiation or chlorination—to ensure the complete inactivation of any residual biological material prior to final release. These measures align with international regulatory frameworks for modified organisms, ensuring that this innovative antiviral approach remains both ecologically safe and commercially viable.

Taken together, our results support the use of genetically engineered live feed organisms as RNAi delivery vehicles in shrimp culture and provide a basis for further evaluation of this approach in aquaculture.

## Data Availability

The raw data supporting the conclusions of this article will be made available by the authors, without undue reservation.
