# Supplementary material for: A transgenic rotifer-based RNA interference approach for antiviral protection against Covert mortality nodavirus in shrimp aquaculture
Source: Front Microbiol. 2026 Jun 10;17:1860691. doi: 10.3389/fmicb.2026.1860691 (PMC13312535; doi:10.3389/fmicb.2026.1860691)
Supplement: Supplementary file 2 [file Table_2.docx]

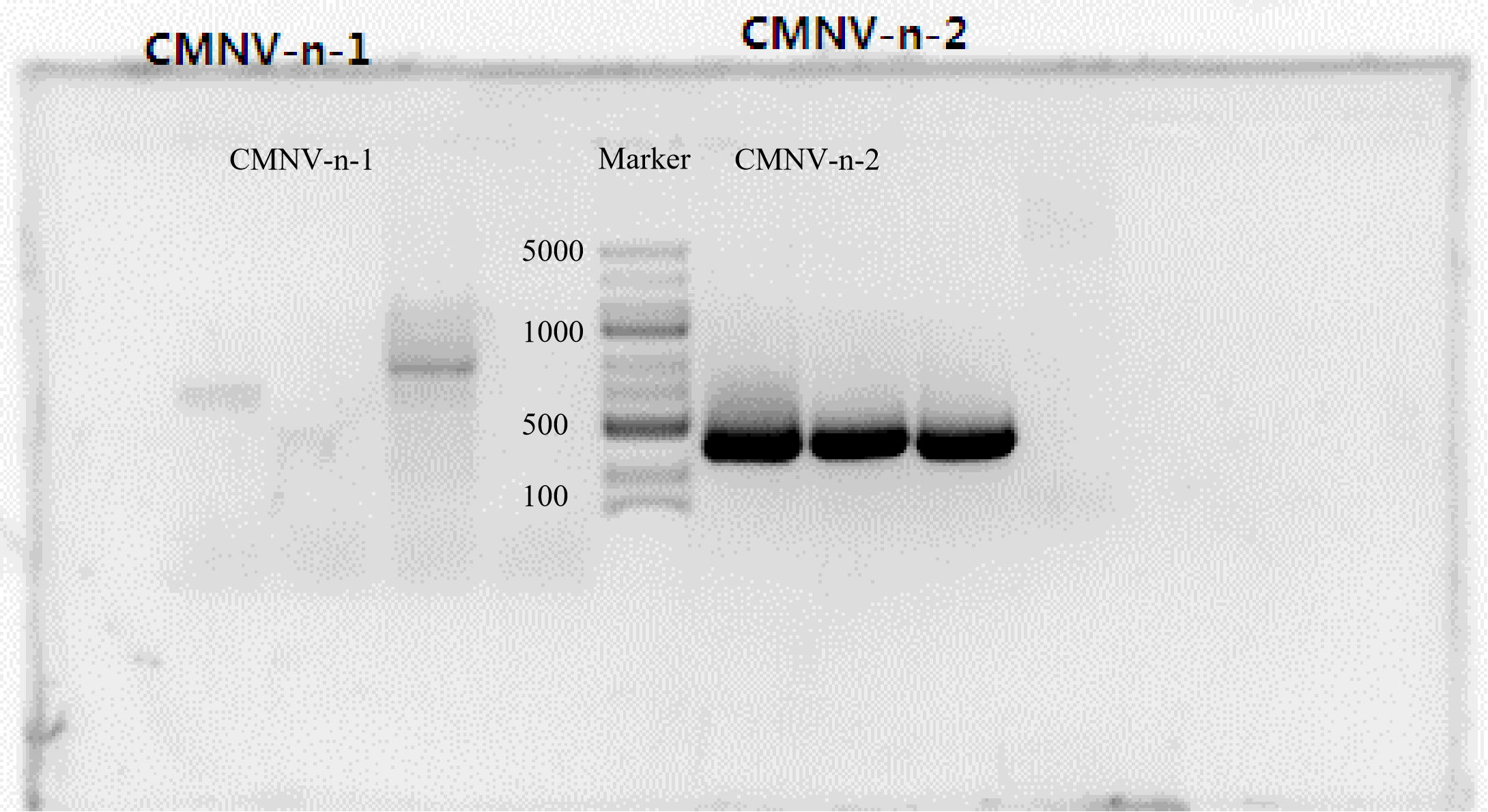


**Figure S1. Original uncropped gel image for RT-nested PCR detection of CMNV.** The figure shows the first-round (CMNV-n-1) and second-round (CMNV-n-2) amplification products for the three *P. vannamei* samples analyzed in this study. Lane M: DNA marker (5000 bp). This original image corresponds to the results presented in Figure 3A.


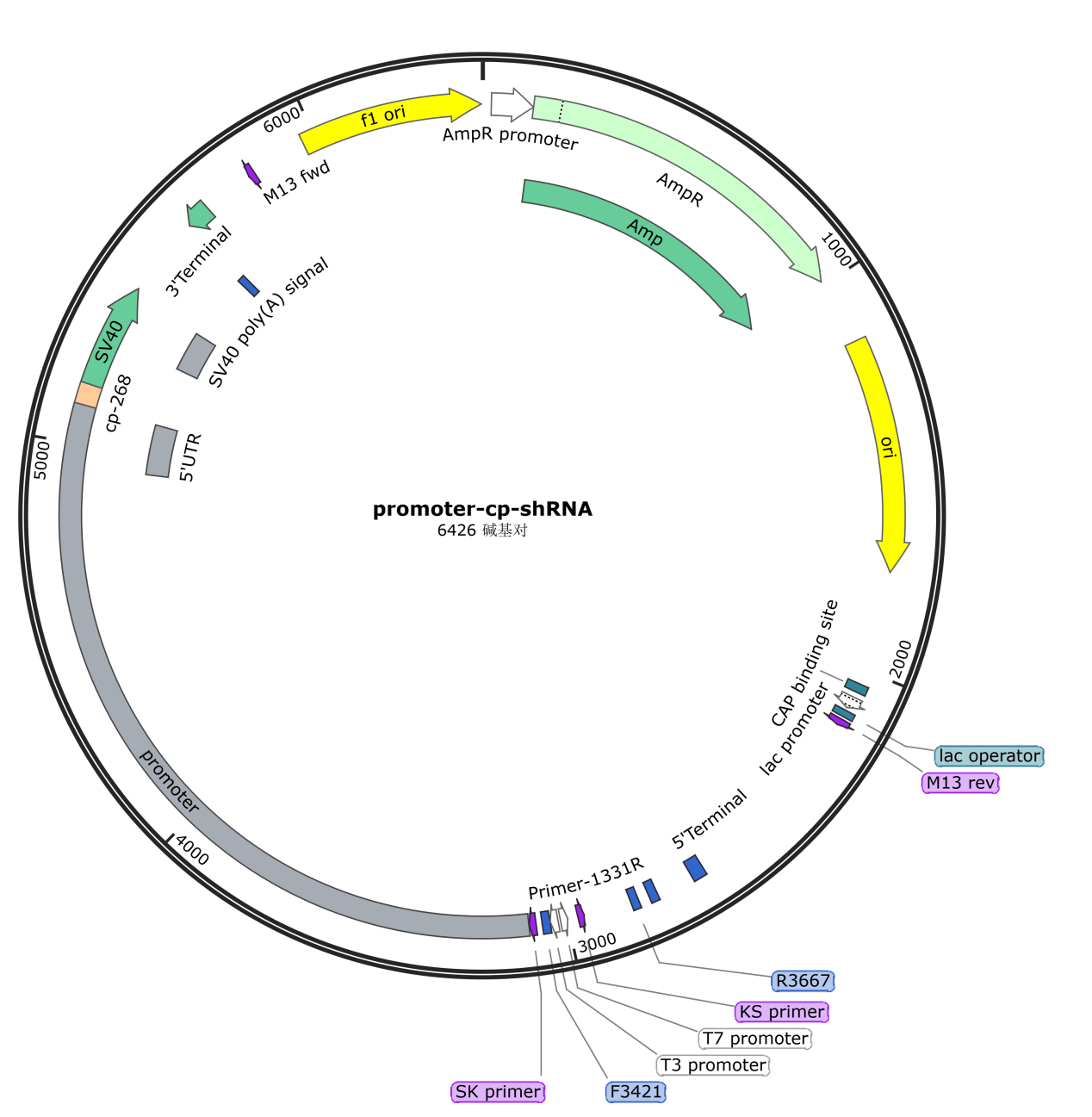


**Figure S2. Map of the piggyBac donor plasmid carrying the *CMNV-cp* shRNA cassette.**
This plasmid was constructed to express a short hairpin RNA targeting the coat protein (cp) gene of CMNV in B. plicatilis. The Cp-268 shRNA cassette was inserted downstream of the selected endogenous B. plicatilis promoter and 5′ UTR, followed by the SV40 poly(A) signal. The expression cassette is flanked by the piggyBac 5′ and 3′ terminal repeat regions, allowing transposase-mediated genomic integration into the rotifer genome. The vector backbone contains bacterial propagation and selection elements, including the ampicillin resistance gene and bacterial origin of replication. The detailed Cp-268 shRNA target sequence, sense strand, loop, antisense strand, and relevant cloning sites are listed in Supplementary Table S1.

**
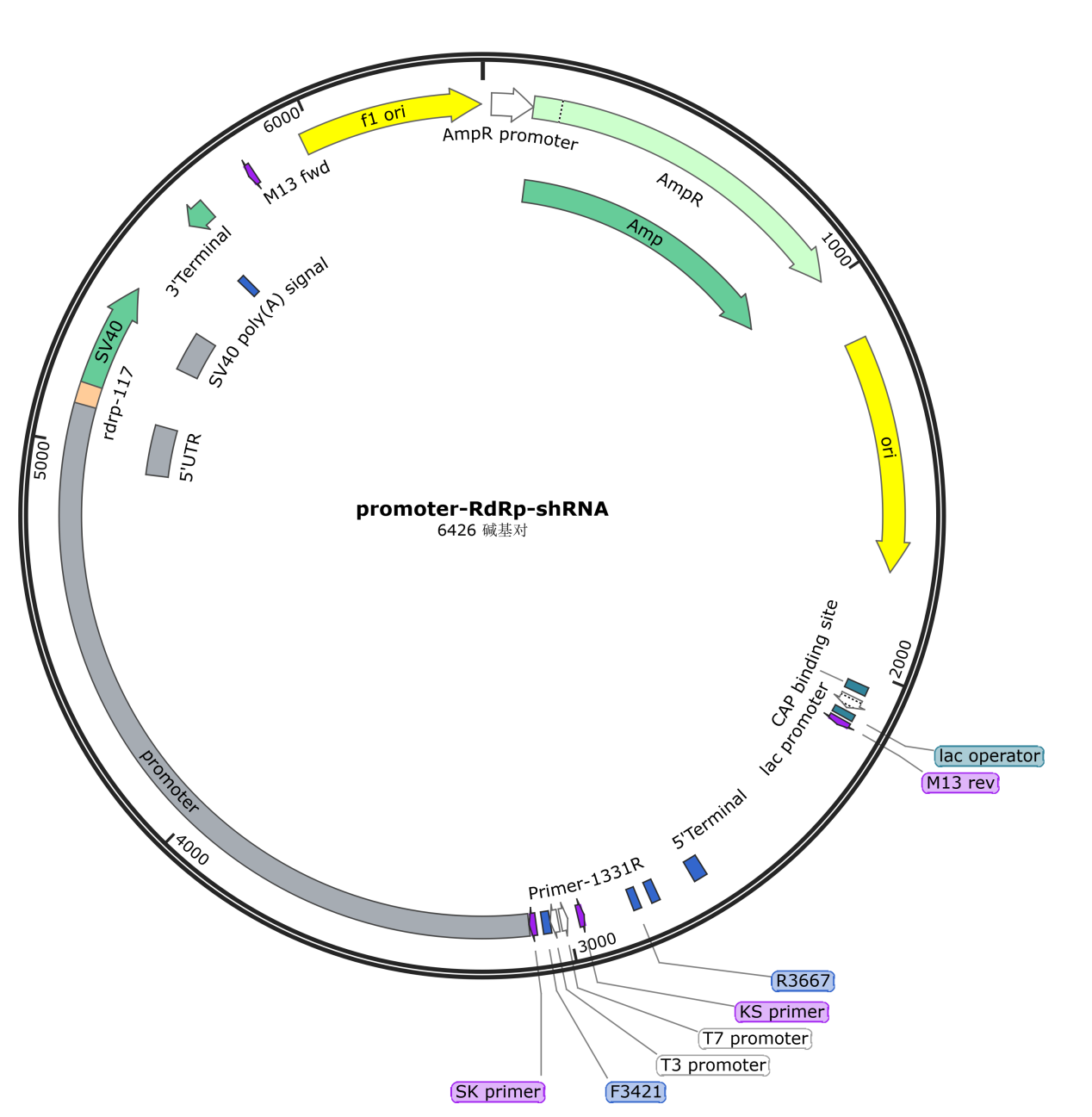
**

**Figure S3. Map of the piggyBac donor plasmid carrying the *CMNV-RdRp* shRNA cassette.**
This plasmid was constructed to express a short hairpin RNA targeting the *RNA-dependent RNA polymerase* (RdRp) gene of CMNV in B. plicatilis. The RdRp-117 shRNA cassette was inserted downstream of the selected endogenous B. plicatilis promoter and 5′ UTR, followed by the SV40 poly(A) signal. The expression cassette is flanked by the piggyBac 5′ and 3′ terminal repeat regions, allowing transposase-mediated genomic integration into the rotifer genome. The vector backbone contains bacterial propagation and selection elements, including the ampicillin resistance gene and bacterial origin of replication. The detailed RdRp-117 shRNA target sequence, sense strand, loop, antisense strand, and relevant cloning sites are listed in Supplementary Table S1.
